# The creation and validation of the Measure of Effective Attributes of Trainers (MEAT)

**DOI:** 10.1186/s13012-017-0603-y

**Published:** 2017-06-02

**Authors:** Meredith R. Boyd, Cara C. Lewis, Kelli Scott, Anne Krendl, Aaron R. Lyon

**Affiliations:** 1 Psychological and Brain Sciences, Indiana University, 1101 East 10th Street, Bloomington, IN 47405 USA; 2 Kaiser Permanente Washington Health Research Institute, 1730 Minor Ave, Seattle, WA 98101 USA; 3 Psychiatry and Behavioral Sciences, University of Washington School of Medicine, 6200 NE 74th Street, Suite 100, Seattle, WA 98115 USA

**Keywords:** Training, Psychometric properties, Exploratory factor analysis

## Abstract

**Background:**

Training is a core component in the implementation of empirically supported treatments, especially in the case of psychosocial interventions targeting mental illness. However, common forms of training are relatively ineffective in producing behavioral changes in providers. Trainers are in a strategic position to influence the success of training, but no research, to our knowledge, has explored whether personal characteristics of trainers (e.g., enthusiasm, charisma) increase effectiveness of training empirically supported treatments in the field of mental health. To address this gap, the current study created a measure of trainer characteristics (the Measure of Effective Attributes of Trainers (MEAT)) and assessed preliminary evidence for its reliability and validity by following gold standard measure development procedures.

**Methods:**

Measure development consisted of three steps: (1) An initial pool of items was generated based on extant literature, input from the target population, and expert input; (2) target users of the measure interacted with the initial item pool to ensure face validity as well as clarity of measure instructions, response options, and items; and (3) a convenience sample viewed training videos and completed the measure resulting from step 2 to establish preliminary evidence of reliability and validity. An exploratory factor analysis was performed on the measure to determine whether latent factors (i.e., subscales of characteristics) underlie the data.

**Results:**

The final solution consisted of two factors that demonstrated preliminary evidence for structural validity of the measure. The first factor, labeled “Charisma,” contained items related to characteristics that facilitate a positive personal relationship with the trainee (e.g., friendly, warm), and the second factor, labeled “Credibility,” contained items related to characteristics that emphasize the qualification of the trainer (e.g., professional, experienced). There was also evidence for face validity, content validity, reliability, and known groups validity of the measure.

**Conclusions:**

The MEAT demonstrated preliminary evidence of key psychometric properties. Future research is needed to further explore and contribute to its psychometric evidence, which could be done in conjunction with measures of trainee knowledge, attitudes towards empirically supported treatments, and evaluations of trainee behavior change to delineate key characteristics of trainers to be leveraged for more effective training.

**Electronic supplementary material:**

The online version of this article (doi:10.1186/s13012-017-0603-y) contains supplementary material, which is available to authorized users.

## Background

Decades of research have culminated in numerous empirically supported treatments (ESTs) for a range of mental health problems. Training has been identified as a core component of implementing ESTs [1]. However, training, as it is most commonly conducted (single exposure didactic events), is typically ineffective in producing trainee behavioral changes [2]. While some strategies increase the effectiveness of training (e.g., consultation [3]), little attention has been given to trainers themselves. Trainers are in a strategic position in which their personal characteristics (i.e., trainee-perceived aspects) could influence training success. Given consistent findings that people automatically and rapidly form impressions of others and those impressions profoundly impact behavior [4], this is a potentially productive avenue for improving training.

Numerous studies articulated the need to study trainer characteristics (e.g., [5, 6]), and across disciplines, some have sought to disentangle the effect of the trainer or supervisor (who often operates in a similar capacity as the trainer). For example, in the human resource literature, Cooper found trainer characteristics (*relaxed*, *tranquil*, *self-sufficient*) were related to subsequent improved work performance by trainees [7]. Characteristics of supervisors such as being *motivational* or *intellectually stimulating* have been shown to influence motivation and goal commitment, which in turn increase job performance [8]. In the medical and mental health supervision literature, characteristics of supervisors, such as being *charismatic* or *likable*, have been shown to influence supervisees’ satisfaction with training [9]. These studies suggest that trainers may be in a critical position to influence trainees’ attitudes and motivation, factors that contribute to intention to carry out behavior [10].

Despite the need for effective training of the workforce in ESTs, no research has specifically explored the impact of trainer characteristics on trainee outcomes in the field of mental health. As a first step to addressing this gap, the current study aimed to (1) use recommended procedures for measure development, informed by input from existing training literature across multiple disciplines, to create the Measure of Effective Attributes of Trainers (MEAT) and (2) assess the effects of trainer characteristics on intention to use skills learned in training. Intention was chosen as it has been shown to be a strong predictor of subsequent behavior [10].

## Methods

Measure development consisted of three steps. Step 1: an initial item pool was generated based on extant literature and expert input. Step 2: target users of the measure interacted with the item pool to ensure face validity, and clarity of measure instructions, response options, and items. Step 3: a convenience sample viewed training videos and completed the measure resulting from Step 2 to assess preliminary reliability and validity.

### Step 1: generate items

The literature review, conducted in the fall of 2014 on Google Scholar, targeted mental health, medical, human resource, and education literature that referenced trainer or supervisor characteristics. Though not a systematic review, the literature was searched until a point of redundancy was reached, meaning, no new characteristics were emerging. An example search string was “trainer AND qualities OR characteristics OR traits AND medical.”

Independently, a list of trainer characteristics was compiled from semi-structured interviews and online surveys administered to leading training experts (*N =* 6) and students (*N* = 14) in Ph.D. or master’s programs in mental health with a clinical training component. Since the aim of this step was to create a comprehensive list of characteristics, items were included regardless of whom or how many times they were listed. Trainers had extensive training experience (average of 21 years as a trainer) and were renown in the field (e.g., published training manuals, trained hundreds of clinicians each year). All trainers were Caucasian, and 50% were female. Consistent with our intention to develop a measure that would cut across theoretical orientations, trainers represented a range of approaches. Student participants were selected due to their exposure to extensive educational, professional, and practicum training experiences. Trainees were predominantly female (85.7%) and Caucasian (78.6%). Trainees had received training in a number of theoretical orientations.

### Step 2: evaluate item clarity and face validity

A separate group of trainees (*N* = 8) interacted with the preliminary item pool. Participants included students in Ph.D. or master’s programs with a mental health clinical training component who were predominantly female (87.5%) and Caucasian (87.5%) and had training in a variety of theoretical orientations. The purpose of this step was to ensure face validity of the measure for the target user (trainees) and improve instruction, item and response option clarity, and utility. In the first round, four participants viewed a 15-min video of an individual delivering mental health training and completed the measure as if the person featured in the video was their trainer. The first author used probing techniques to elicit feedback about item wording, directions, measure format, and subject matter [11]. The measure was revised based on trainee feedback. In the second round, another four participants watched the video, completed the measure, and provided feedback; the measure was revised. Finally, revisions were made per feedback from three measure development experts (identified through professional contacts).

### Step 3: establish preliminary evidence of validity and reliability

The resulting item pool was administered to undergraduates at Indiana University who were enrolled in an introductory psychology course and participated in exchange for partial course credit. Participants were female (65.4%), Caucasian (79%), and in their freshman year in college (66.7%). Each participant viewed two of four possible videos of the same trainer delivering brief trainings on two different mental health topics. For each training topic, two videos were generated that either emphasized the trainer’s credibility and professionalism (hereafter called “professional” trainer) or her approachability and relatability (hereafter called “personable” trainer). In the professional trainer videos, the trainer introduced herself as “Dr.,” referenced her own professional experiences with the topic, and was concise when delivering training. In the personable trainer videos, the trainer introduced herself as a fellow graduate student, referenced personal stories, and made jokes when delivering the training. The video scripts were written and performed by members of the research team (MB and CCL).

Before and after viewing each video, participants completed a measure, constructed using Ajzen’s manual for creating a Theory of Planned Behavior (TPB) Questionnaire [12] that assessed their intention to use the skill learned during the training session. This manual is one of the most widely used approaches for constructing a measure of intention. In the present study, the measure demonstrated good to excellent internal consistency across each version (Cronbach’s coefficient *α* .84 to .91). After viewing each video, the participants completed the MEAT.

This experiment was a between-subject 2 × 2 factorial design (see Table 1). This design was chosen to see if the MEAT was sensitive to the differences in characteristics that trainers expressed (known groups validity discussed below).

**Table 1:** 2 × 2 within and between factorial design for step 3

	Mindfulness	Thought Record
Personable	Mindfulness Personable	Thought Record Personable
Professional	Mindfulness Professional	Thought Record Professional

*Note*: Each participant viewed two training vignettes. Vignettes were counterbalanced for topic and trainer type

### Statistical analyses

#### Structural validity

All analyses were conducted in SPSS (unless otherwise indicated); tests were two-tailed with *p* values set at .05. An exploratory factor analysis (EFA) was performed using data from the measure completed after the first video participants watched to determine the latent factors (i.e., subscales of characteristics) that underlie the data [13]. Kaiser-Meyer-Olkin (KMO) measure and Bartlett’s Test of Sphericity both suggested the suitability of performing an EFA [14]. A principal axis factoring analysis was selected with an oblique Promax rotation. Parallel analysis was carried out to determine the number of factors to retain using *R*.

According to de Winter, Dodou, and Wieringa, sample size, number of factors retained, number of items, and factor loading contribute to factor recovery [15]. To maximize the reliability of factor recovery, items were retained only if they loaded near .60 on the primary factor.

#### Reliability

Cronbach’s coefficient *α* was calculated for the derived subscales to assess internal consistency.

#### Known groups validity

Each participant viewed a video of a personable trainer and a video of a professional trainer in counterbalanced order and completed the MEAT after watching each video. Two paired-samples *t* tests were used to determine if the MEAT subscale scores were sensitive to trainer differences. Subscale scores were the mean of individual ratings for items.

#### Intention

A paired-samples *t* test was used to determine if trainer type was associated with change in intention to use skills learned during training. Data were collapsed across trainer types for the *t* test.

## Results

### Step 1: generate items

**Fig. 1:**
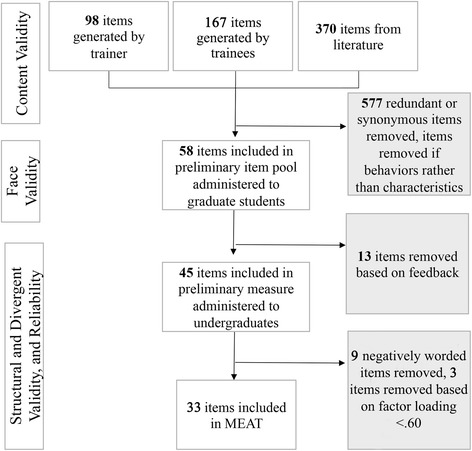
Flow chart of measure items included in MEAT

Trainers generated 98 characteristics, trainees generated 167 characteristics, and 370 characteristics emerged from a review of human resource, education, and medical literature. Repetitive items and items that reflected behaviors rather than characteristics were removed. With expert input, 58 unique characteristics were retained (Fig. 1).

### Step 2: evaluate item clarity and face validity

Based on feedback from two groups of trainees who interacted with the initial item pool, another 13 items were removed because they were redundant with other items or lacked clarity.

### Step 3: establish preliminary evidence of validity and reliability

#### Structural validity

Bartlett’s Test of Sphericity was significant (*x* ^2^(528) = 2564.92, *p* < .001) and the KMO measure was “meritorious” (KMO = .89) [13]. The final solution consisted of two factors with 24 items loading on to the first factor and 9 items loading on to the second factor. The first factor, labeled “Charisma,” contained items related to characteristics that facilitate a positive personal relationship with the trainee, and the second factor, labeled “Credibility,” contained items related to characteristics that emphasize the credibility and qualification of the trainer. The Charisma subscale accounted for 52.97% of the variance and the Credibility subscale accounted for 11.30% of the variance, for a total of 64.27% of variance explained. Table 2 provides the factor loadings, and Additional file 1 includes the MEAT with instructions.

**Table 2:** MEAT subscale and item means, standard deviations, eigenvalues, Cronbach’s alpha, and exploratory factor analysis loading

	Mean	SD	EV	*α*	Factor 1	Factor 2
1. Charisma			17.48	.98		
Warm	2.61	1.23			*.98*	−.17
Caring	2.80	1.18			*.89*	−.01
Considerate	2.93	1.20			*.89*	.01
Enthusiastic	2.51	1.35			*.87*	−.18
Approachable	2.87	1.28			*.87*	−.06
Friendly	2.96	1.27			*.86*	−.10
Supportive	2.97	1.22			*.86*	.05
Passionate	2.59	1.20			*.86*	.00
Likeable	2.61	1.19			*.86*	−.02
Sociable	2.78	1.20			*.84*	−.02
Entertaining	2.00	1.06			*.80*	−.07
Engaging	2.57	1.23			*.79*	.00
Patient	2.95	1.09			*.76*	.03
Motivational	2.57	1.15			*.75*	.00
Able to listen	2.83	1.14			*.75*	.09
Humorous	1.96	1.01			*.75*	−.18
Empathetic	2.75	1.07			*.74*	−.07
Flexible	2.42	1.10			*.74*	.05
Accessible	2.86	1.17			*.73*	.15
Humble	3.05	1.09			*.71*	.08
Respectful	3.30	1.19			*.67*	.12
Open to criticism	2.82	1.15			*.63*	.11
Intellectually stimulating	2.54	1.10			*.61*	.22
Trustworthy	3.11	1.05			*.61*	.31
2. Credibility			3.73	.91		
Prepared	3.43	1.08			−.30	*.87*
Intelligent	3.70	0.95			−.03	*.84*
Professional	3.37	1.08			−.11	*.83*
Expert	3.17	1.01			.00	*.71*
Organized	3.41	1.10			−.13	*.71*
Experienced	3.43	1.06			.09	*.70*
Knowledgeable	3.75	0.91			.16	*.70*
Skillful	3.16	0.94			.25	*.57*
Communicates effectively	3.24	1.14			.31	*.54*

Factor loadings above .60 appear in italics

*Note*: *N* = 76, *SD* standard deviation, *EV* eigenvalue, *α* Cronbach’s alpha

#### Reliability

The internal consistency of the MEAT subscales was excellent: Charisma, *α* = .98; Credibility, *a* = .91.

#### Known groups validity

We assessed whether participants’ ratings of the trainer on the subscale items differed between the personable and professional trainer conditions. As predicted, the Charisma score was higher for the personable, but the Credible score was higher for the professional trainer: *t*(70) = −7.95, *p* < .001 and *t*(72) = 2.52, *p* = .014, respectively (see Table 3).

**Table 3:** Paired *t* test results comparing MEAT subscale scores when viewing personable or professional trainers

Subscale	Trainer type viewed	Mean	SD	*t*	*df*	*p*
Charisma Subscale Score	Personable	3.46	.70	−7.95	71	.00
	Professional	2.39	.88			
Credible Subscale Score	Personable	3.04	.79	2.52	73	.01
	Professional	3.37	.86			

#### Intention

To explore the role of trainer characteristics in training effectiveness, we examined participant self-report of intention to use skills learned in training, dependent on trainer type. Paired-samples *t* tests indicated that participants had a significantly higher intention to use the skills learned when the trainer was personable (*M* = 2.97, SD = 10.15) rather than professional (*M* = −1.11, SD = 11.35), *t*(75) = 2.42, *p* = .02. There was no significant difference in intention for topic, *t*(75) = .92, *p = .*36.

## Discussion

Preliminary evidence suggests that the MEAT is a valid and reliable measure of trainer characteristics. The structural validity revealed two factors reflecting charismatic and credible characteristics of trainers. Results also suggest that trainer characteristics, specifically those related to trainer charisma, are associated with a trainee’s intention to apply the skills they learn in training which is important given the ability of intention to predict subsequent behavior [10].

The resulting factors of trainer characteristics readily map onto published literature promoting the effectiveness of clinical supervision in mental health settings. The Supervisor Rating Form (SRF) [9] was adapted from a measure of counselor characteristics to assess the extent to which the supervisor expresses characteristics related to the components of Strong’s Social Influence Model: Attractiveness, Expertise, and Trustworthiness [16]. The SRF Attractiveness subscale shares items with the MEAT Charisma subscale (likeable, friendly, warm, and sociable). The SRF Expertise subscale shares items with the MEAT Credibility subscale (experienced, expert, and prepared). Although the MEAT subscales were not created to map these SRF subscales, items from the SRF subscales were included in the initial item pool for the MEAT. The similarities between these two measures suggest that trainers may contribute to training effectiveness by exerting positive social influence over trainees. However, items from the MEAT were specifically generated by mental health trainers and trainees and therefore reflect unique needs in mental health training.

### Limitations

Results for the structural validity and reliability were obtained from a convenience sample of undergraduate psychology majors rather than a representative sample of mental health trainees. However, convenience samples are commonly used in the first stages of measure development since content-specific knowledge is not necessary to interact with a measure (e.g., [17]). Some conventional wisdom would suggest the present study was underpowered for the EFA (i.e., five to ten participants per item [18]). However, both the KMO measure and Bartlett’s Test of Sphericity suggested the appropriateness of performing EFAs. Further, based on the number of factors (2), number of items (36), and factor loading (average of .78 on first factor and .72 on the second factor), de Winters et al. state that a sample as low as 34 is appropriate [15]. Google Scholar was used to conduct the literature review even though this database has been criticized for its lack of transparency and personalization feature. However, to ensure rigor of our approach, two research assistants conducted initial searches, collected relevant citations, and provided article summaries. The first author reviewed full-text articles and did targeted searches for additional useful citations. Although the review was not a systematic review, the literature was searched until a point of redundancy was reached, meaning no new characteristics were emerging. Input from expert trainers and trainees also ensured that relevant items were not missed.

### Future directions

To further establish evidence for the psychometric properties of the MEAT, the factor structure will be confirmed with a large, representative sample of mental health trainees. Convergent and divergent validity of the measure will also be assessed. Measures of effective training behaviors should be compared to the MEAT to establish divergent validity as the MEAT relates to effective characteristics and not behaviors. The MEAT will be administered to mental health trainees after training along with measures of intention (e.g., Theory of Planned Behavior), attitudes, and observation of subsequent behavior change to determine if trainer characteristics are associated with any of these desirable training outcomes. If certain trainer characteristics are found to be associated with desired training outcomes, the MEAT could then be used to select or train trainers to be more effective in their role. Specifically, there is a growing interest in “train-the-trainer” (TTT) initiatives in which experts in specific ESTs identify and provide instruction to staff members (e.g., clinicians) who then provide training to others in their organization [19]. The MEAT could inform both characteristics fostered within clinicians being trained to train others in their organization and to select among professional trainers who conceivably would have been evaluated using the MEAT in the past training and chosen based on the characteristics they possess. However, before the MEAT can be used for selection purposes, strong evidence for predictive validity of the measure must first be established.

## Conclusions

In order to administer the best training possible to maximize this critical component of an implementation process, the role of the mental health trainer needs to be acknowledged and understood. The MEAT has demonstrated preliminary evidence as a psychometrically strong measure of trainer characteristics to be used to unravel the impact of the trainer in training effectiveness.

## Additional file

Additional file 1: Formatted MEAT with directions. (PDF 32 kb)

## Abbreviations

EFA: Exploratory factor analysis
KMO: Kaiser-Meyer-Olkin
MEAT: Measure of Effective Attributes of Trainers
SRF: Supervisor Rating Form
TTT: Train-the-trainer
