# Supplementary material for: The creation and validation of the Measure of Effective Attributes of Trainers (MEAT)
Source: Implement Sci. 2017 Jun 2;12:73. doi: 10.1186/s13012-017-0603-y (PMC5457566; doi:10.1186/s13012-017-0603-y)
Supplement: Additional file 1: — Formatted MEAT with directions. (PDF 32 kb) [file 13012_2017_603_MOESM1_ESM.pdf]

Measure of Effective Attributes of Trainers (MEAT)

Please rate your trainer as accurately as possible along each of the characteristics listed. Choose the number indicating the extent to which that characteristic describes your trainer using the scale below.

1	2	3	4	5
Very slightly/not at all	A little	Moderately	Quite a bit	Extremely

_____Experienced

______Likeable

______Sociable

______Friendly

______Entertaining

______Prepared

______Skillful

______Caring

______Open to criticism

______Warm

______Passionate

______Trustworthy

______Respectful

______Able to listen

______Enthusiastic

______Engaging

_______Organized

______Supportive

______Approachable

______Accessible

______Knowledgeable

______Motivational

______Expert

______Humorous

______Flexible

______Considerate

______Professional

______Humble

______Intelligent

______Patient

______Empathetic

______Intellectually stimulating

______Communicates effectively
